# Role of Mobile Health and Wearable Devices in Cardiovascular Disease Prevention: A Comprehensive Review

**DOI:** 10.7759/cureus.83673

**Published:** 2025-05-07

**Authors:** Owais M Ahmad, Ruba Ibrahim, Daniella I Odunsi, Mahdi Mohammed, Bobby George Mathew, Mohamed Touny, Bhupinder S Grewal, Shubham Bhanot, Neeraj Bodapati, George Pandarakalam Thomas, Shreyas NV, Lalain Masood, Zoya Morani

**Affiliations:** 1 Internal Medicine, Charles University, Hradec Kralove, CZE; 2 Medicine and Surgery, University of Medical Sciences and Technology (UMST), Khartoum, SDN; 3 Internal Medicine, Barts and the London School of Medicine and Dentistry, Queen Mary University of London, London, GBR; 4 School of Medicine, University College Dublin, Dublin, IRL; 5 Anaesthesia, Chris Hani Baragwanath Academic Hospital, Johannesburg, ZAF; 6 Cardiology, National Heart Institute, Giza, EGY; 7 Cardiovascular Service Line, Morristown Medical Center, Morristown, USA; 8 Acute Medicine, Cumberland Infirmary, Carlisle, GBR; 9 Internal Medicine, Alluri Sitarama Raju Academy of Medical Sciences, Eluru, IND; 10 Internal Medicine, Dorset County Hospital, Dorchester, GBR; 11 General Medicine, Shirdi Sai Hospital, Bengaluru, IND; 12 Dermatology, Bahria University Medical and Dental College, Karachi, PAK; 13 Family Medicine, Washington University of Health and Science, San Pedro, BLZ

**Keywords:** artificial intelligence (ai), cardiovascular disease (cvd), cardiovascular disease prevention, cvs, mobile health (mhealth), smartphones, smartwatches, wearable technology

## Abstract

Cardiovascular disease (CVD) is a leading cause of death worldwide. A key area of interest in CVD prevention is novel digital health technologies, primarily mobile health (mHealth) applications and wearable devices, that are rapidly transforming the methods of preventing and managing CVD. Studies have shown the success of smartphone applications, such as the RITMIA app (Heart Sentinel™, Parma, Italy), in successfully detecting atrial fibrillation (Afib) compared to a classic 12-lead electrocardiogram (ECG). mHealth devices should integrate these factors, based on artificial intelligence (AI) and driven by chatbots, to encourage patients to use technology through interactive, real-world, motivational, and timely feedback. Data from mHealth clinical research indicate improved medication adherence, weight control, and self-care among patients. This review highlights mHealth and wearable devices in CVD prevention, providing foresight into cardiovascular health conditions through continuous monitoring, early detection, and improved patient engagement. Additionally, it examines challenges, including ethical, regulatory, and accessibility issues, that need to be addressed before their widespread adoption. In the future, the priority must be integration with healthcare systems and equitable access. A thorough search was conducted using reputable databases such as Scopus, PubMed, and Google Scholar. Articles from 2015 to 2025, along with an article from 2002 published in reputable peer-reviewed journals, were analyzed and contextually used. We also refined our search terms and used high-quality English articles to achieve this.

## Introduction and background

The objective of this review is to scrutinize, assess, and evaluate the role of mobile health (mHealth) and wearable devices in preventing cardiovascular disease (CVD). Additionally, it aims to determine the validity and accuracy of cardiovascular biomarkers detected by wearable sensors. The review will also address usability, privacy, long-term engagement challenges, and future directions and innovations in digital cardiovascular health. This review further aims to inform future research, policy, and clinical applications.

CVD is the number one cause of death worldwide and includes a wide range of diseases, such as ischemic heart disease (IHD), peripheral vascular disease (PVD), arrhythmias, and cerebrovascular disease. Once thought to be a condition primarily affecting men, one in three women experiences some form of heart disease in their lifetime; moreover, IHD alone is responsible for 16% of deaths globally. In the United States alone, heart disease led to 702,880 deaths in 2024 and incurs annual medical costs of up to $33 billion, with most of these deaths occurring in low- and middle-income countries [1,2].

The main risk factors for CVD are both behavioral and metabolic. Behavioral risk factors include smoking, physical inactivity, and an unhealthy diet. These behavioral risk factors, in the long term, can lead to metabolic issues such as obesity, hypertension, dyslipidemia, and diabetes. Furthermore, non-modifiable risk factors include age, race, sex, and family history [2].

Early detection of CVD can prevent its progression to more severe conditions, such as myocardial infarction (MI), heart failure, and other cardiovascular events [1]. In this regard, novel digital health technologies - primarily mHealth applications and wearable devices - are rapidly transforming the methods of preventing and managing CVD [3]. These include using mobile devices, such as smartphones and tablets, to provide health interventions, promote patient engagement and adherence, and facilitate remote monitoring [2,3]. Consequently, smart devices like smartwatches, fitness trackers, and electrocardiogram (ECG) monitors have gained viral traction, as they can track real-time physiological parameters such as heart rate, physical activity, and sleep patterns [4].

Such technologies offer an anticipatory method for the awareness and management of healthcare. Their combination with artificial intelligence (AI) and cloud-based platforms promotes real-time data analysis, remote consultation, and timely medical interventions [3]. Hence, mHealth and wearable devices may bridge the gap between the paternalistic approach to healthcare and self-management strategies [5].

Although mHealth has a growing variety of applications, these solutions still face challenges. Among these are data accuracy, user adherence, regulatory issues, and integration into clinical settings. The effectiveness of mHealth interventions in CVD prevention has already been investigated in a number of studies, yet a review focusing on their impact, limitations, and future directions is warranted [6].

## Review

Wearable mHealth devices: methods, applications, and emerging trends

Wearable mHealth devices represent a game-changer for the healthcare sector because they provide continuous monitoring and real-time data gathering. Wearable technology devices, such as smartwatches, fitness trackers, and biosensors, are gaining widespread popularity, particularly those equipped with advanced sensors that can accommodate physiological parameters such as heart rate, blood pressure, glucose, and much more [7,8]. This information can be sent wirelessly to healthcare professionals, who can supervise from a distance and intervene if needed [9]. Wearable ECG devices, for example, can detect arrhythmia and other underlying heart conditions, providing critical clues for doctors to catch them early and intervene promptly [10,11]. These come in different forms: they can be worn as ECG patches or chest straps, which are the most accurate, or even with advanced smartwatches and fitness trackers. Choosing which device to use often depends on the patient's preference and special needs. Moreover, continuous glucose monitors worn on the back of the upper arm, abdomen, thigh, and lower back have provided patients with continuous trends in blood glucose levels, making them an essential tool for modern diabetes management and effectively lowering the frequency of clinic visits. Moreover, maintaining a regular blood glucose level will, in turn, decrease the risk of cardiovascular events [9].

In addition, implementing algorithms using AI and machine learning also provides an added benefit to these devices, as it offers individualized health suggestions and predictive insights [10]. Wearable mHealth devices should be well-positioned to increase their efficacy and accuracy. These devices can be worn on various body parts, including the wrist, near the chest, or attached directly - for example, as an adhesive patch [7]. Convenience and ease of use are two key reasons why wrist-worn devices, such as smartwatches and fitness trackers, are so popular [9]. Wearable technologies also track daily habits, sleep patterns, and feelings, offering a wide-ranging picture of a person's overall health [10]. Chest-worn devices (e.g., ECG monitors) often give more accurate cardiac measurements since they are relatively close to the organ in question. Similar to microfluidic patches, adhesive patches allow for the continuous measurement of biomarkers in the body, which has applications in chronic disease diagnosis and management. The device selection and placement are based on the type of health metrics monitored and the user, ensuring optimal performance and comfort [9].

**Figure 1:**
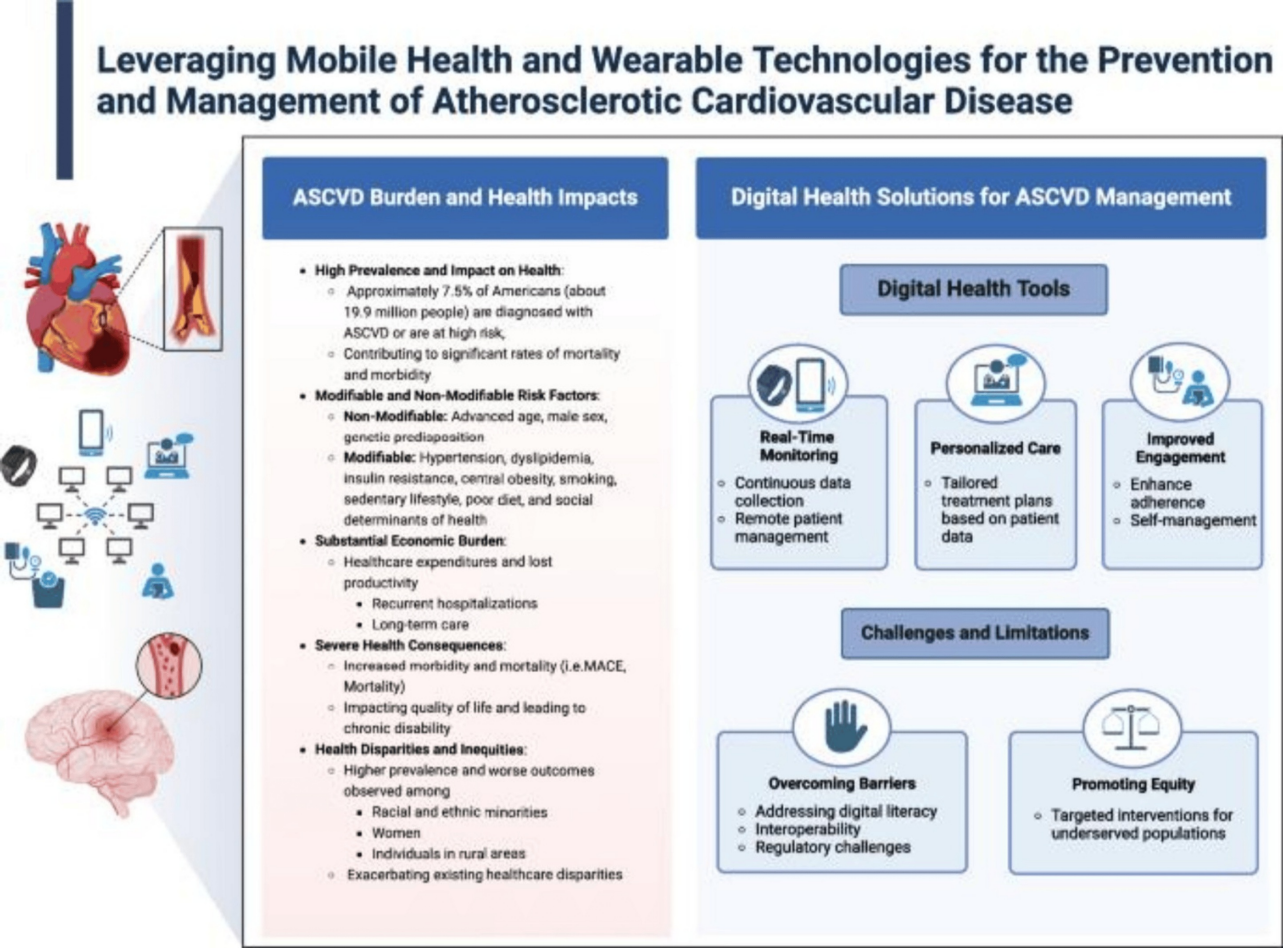
Infographic highlighting role of mHealth and wearable technologies This figure illustrates the role of mHealth and wearable technologies in managing ASCVD, addressing its burden, risk factors, and digital solutions, as well as key challenges such as accessibility and digital literacy. Image credit: Permission has been obtained to reproduce the images from the publishers [13]. mHealth, mobile health; ASCVD, atherosclerotic cardiovascular disease

Moreover, mHealth technology has become increasingly prominent for the prevention of CVD in the past few years. The most prominent trend includes the combination of wearable devices with mHealth applications, available for real-time data collection and continuous monitoring [12]. Additionally, new studies have observed that through mHealth attempts, augmented with wearable technology, lifestyle modifications to promote healthy behaviors, real-time physiological monitoring, and early prevention of atherosclerotic cardiovascular disease (ASCVD) through key risk factor management (i.e., metabolic syndrome and sedentary lifestyle) can be undertaken [13]. Figure 1 outlines the role of mHealth in ASCVD prevention and management.

Applications employing Bluetooth chest straps can identify atrial fibrillation (Afib) with a comparably high accuracy when measured against standard systems of ECG monitoring [14]. It has been shown that smartwatches' ECG capabilities for the early detection of arrhythmia have high sensitivity [15]. These capabilities are further expanded with machine learning algorithms and AI techniques that offer personalized health advice and predictive analytics for targeted interventions.

Studies have shown that patients using wearables and smartphone applications improve their weight control, quality of life, and medication compliance in clinical trials [8]. Patient-centered mHealth interventions have been shown to improve self-care (detailed management) in heart failure patients, especially with respect to their medication adherence and health monitoring [16].

On the horizon, there are wirelessly connected ECG-integrated fabrics, voice assistant interfaces, and wearable stethoscopes that enable continuous remote monitoring [1,17,18]. Notwithstanding their potential benefits, widespread adoption is limited by high costs, technological constraints, and disparities in digital literacy [19]. In order to best leverage the impact of mHealth strategies for preventing CVD, future efforts must focus on developing accessible solutions.

Wearable device prevention of Afib

Afib has several risk factors, such as hypertension, obesity, and a sedentary lifestyle. Providing patients with an mHealth device may encourage them to lead a better, more active, and healthier lifestyle. On the other hand, this doesn’t target the major obstacle of taming one’s behavior, which may not be improved by having a smartphone application [20].

Recent mobile cardiac outpatient telemetry (MCOT) systems, like NUVANT Mobile Cardiac Telemetry (Corventis, Inc., San Jose, CA, USA), provide a low-profile PiiX sensor that enables a wireless arrhythmia monitor, requiring up to seven consecutive days to collect real-time ECG data. Accepted patient compliance has been shown to be associated with this device. The use of mHealth devices allows remote physicians to monitor patients and provide immediate action at the onset of a serious cardiac arrhythmia [21].

**Figure 2:**
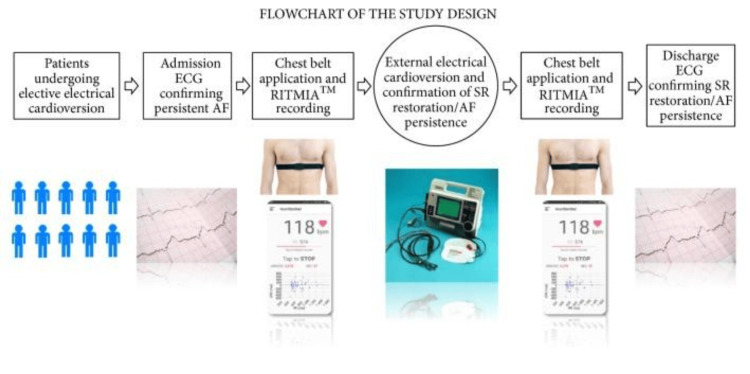
Flowchart of study design This figure illustrates an example of a chest-belt heart rate monitor sensor, similar to the ones used in the study, and an example of the RITMIA app interface for detecting various arrhythmias. Image credit: This figure has been adapted from an open-access article [14], distributed under the terms of the Creative Commons Attribution-NonCommercial-NoDerivatives 4.0 License (CC BY-NC-ND 4.0) (https://creativecommons.org/licenses/by-nc-nd/4.0/).

Various novel applications, such as the RITMIA app (Heart Sentinel™, Parma, Italy) on smartphones, accompanied by a chest strap, involve consumer-grade Bluetooth that provides continuous and automated heart rate monitoring to detect Afib. The study showed that RITMIA app interpretations were successfully accepted for detecting Afib in comparison with a classic 12-lead ECG [14]. Figure 2 shows the study design with an example of the RITMIA app.

Wearable cardioverter defibrillator (WCD)

In industrialized nations, the most common cause of cardiovascular death is sudden cardiac death (SCD). The WCD device (LifeVest; ZOLL Medical Corporation, Chelmsford, MA, USA) has been in use since 2001 as an emergency therapy for “temporary bridging” in patients at significantly high risk of SCD. The WCD consists of a vest garment with straps containing ECG and defibrillator electrodes. It continuously records and interprets two-channel ECG signals. Several forms of alarms - acoustic, tactile, and visual - are triggered to alert the patient before the initiation of a shock when a life-threatening arrhythmia is detected. By pressing the response button, the patient has the opportunity to delay the shock if still conscious. If the patient is unable to press the response button, indicating loss of consciousness, the WCD will deliver a shock, which may be repeated up to five shocks [22].

mHealth and controlling heart failure

A recent study has shown improvement in heart failure self-care through a system of SMS text messages designed based on each person’s criteria. The intervention was conducted using a patient-centered mHealth platform, “iCardia4HF,” which involves three different apps: Fitbit, Heart Failure Health Storylines, and Withings. The study showed a significant difference in heart failure self-care in the intervention group compared to the control group. Major differences were found in aspects of health beliefs about medication adherence, self-efficacy, and self-monitoring adherence. The intervention group showed no adverse incidents [16].

mHealth and uncontrolled hypertension

In a randomized controlled trial, individuals with uncontrolled hypertension who began using a wearable device connected to a smartphone application showed better progression in several aspects - such as quality of life, weight control, and medication compliance - but no difference in systolic blood pressure (SBP) compared with controls [23].

Another randomized controlled trial was carried out to assess MI-BP (Motivational Interviewing for Blood Pressure) through a personal monitoring approach in Black individuals with uncontrolled hypertension. Even though it enhanced patient involvement and compliance, the variation in SBP reduction was not numerically significant compared to routine treatment. This suggests that SBP, influenced by various factors, is not readily modified by mHealth alone but requires broader behavioral or systemic changes [24]. Likewise, another study could not discover an impactful enhancement in sustained monitoring participation or SBP using WhatsApp-based programs [25].

Role of wearable technology in the detection of supraventricular arrhythmias (SVAs)

Wearable technology has greatly improved the detection and monitoring of SVAs, due to its offering of continuous, non-invasive cardiac rhythm assessment outside traditional clinical settings [26]. Smartwatches with ECG capabilities and patch-based monitors have demonstrated high sensitivity and specificity in detecting Afib and other SVAs [27]. The Apple Heart Study, one of the most extensive investigations on smartwatch ECG functionality, revealed that wearable technology could identify Afib with reasonable accuracy, potentially reducing the risk of stroke through early intervention [28].

Outside Afib detection, emerging studies explore the potential of wearable ECG monitors to identify other SVAs, such as atrial tachycardia and supraventricular tachycardia (SVT). Wearable devices’ long-term, ambulatory ECG monitoring allows clinicians to detect transient arrhythmias that might go unnoticed in conventional, short-duration ECG recordings [29]. Moreover, integrating AI-driven arrhythmia detection algorithms into wearable devices has further improved diagnostic accuracy and reduced false-positive rates [3].

Despite these advantages, the role of mHealth in detecting SVA presents problems with data filtration and a lack of data exchange with clinicians. Many wearable ECG devices generate large amounts of data, requiring efficient filtering and interpretation systems to differentiate between pathological arrhythmias and benign rhythm abnormalities [27]. Addressing this challenge requires advancements in signal processing and integration with data that clinicians can access.

In the future, researchers and developers should improve algorithm accuracy and enhance clinical interoperability with wearable arrhythmia detection technologies [5]. The continued evolution of wearable ECG monitors, combined with cloud-based analytics and real-time physician alerts, can improve arrhythmia detection and cardiovascular outcomes globally [29]. Table 1 enlists the risk factors, along with the intervention type and its key findings. 

**Table 1:** Outlines the effectiveness of mobile health (mHealth) interventions on cardiovascular risk factors Table credit: This table was created by Daniella I. Odunsi on Microsoft Word, using the information from the references cited [2,5,17,23,30].

Risk Factor	Intervention Type	Key Findings	p-value
Physical Activity	SMS-based reminders, Wearables (Fitbit)	Increased daily step count, improved fitness levels	p = 0.02
Healthy Diet	Mobile coaching apps, mobile health (mHealth) education	Higher adherence to heart-healthy diets	p < 0.01
Medication Adherence	AI-driven reminders, Smart Pill Dispensers	Improved adherence rates in patients with hypertension	p < 0.04
Smoking Cessation	Smartphone Apps (QuitGuide and SmartQuit)	SmartQuit showed higher compliance and quit rates	p < 0.05
Blood Pressure Control	Wearable Blood Pressure Monitors, Remote Coaching	Significant reductions in Blood Pressure among intervention groups	p < 0.03

Patient engagement and adherence

Effective patient engagement is vital to mHealth interventions aimed at CVD prevention. Studies have demonstrated that mHealth applications, particularly those integrating gamification, personalized coaching, and automated reminders, significantly improve adherence to lifestyle modifications and medical treatment [30]. Using wearable devices, like smartwatches and fitness trackers, encourages more physical activity through instantaneous feedback and motivational prompts, resulting in long-term behavior changes [31]. Patients who utilize mHealth platforms that offer interactive features, such as chatbots capable of delivering medication reminders and AI-driven health coaching, are consistently shown in numerous studies to adhere more closely to prescribed health interventions [17].

Furthermore, studies demonstrated that smartphone applications, which incorporate telemedicine aspects and remote coaching, positively affect medication adherence, as well as patient self-efficacy over cardiovascular risk factors [2]. Moreover, wearable devices are equipped with biosensors (e.g., watches, fitness bands, and loop recorders) that collect intrinsic body data and couple it with their powerful processing capabilities. This may allow for the mapping of individual risk patterns and the creation of prevention and therapeutic models [11]. In addition, studies supporting the use of behavioral science techniques in mHealth platforms, such as self-monitoring and goal setting, aim to improve patient adherence and long-term engagement [32].

However, persistent factors like technology fatigue, privacy concerns, and socioeconomic factors may hinder the success of mHealth interventions across diverse communities [33]. This can be further complicated by low digital literacy and disparities in access to smartphones and wearable devices, especially among older adults and those from low-income communities [34].

The challenges presented by the above studies suggest that future research should aim to take more tailored, patient-centered approaches, maximizing engagement with these cardiovascular prevention strategies and ensuring long-term adherence [35]. This is inclusive of designing mobile prescribing in the adaptive intervention framework, applying AI in the personalization approach, and exploiting real-time physiological data to guide personalized feedback and intervention. Furthermore, more intensive investigations on hybrid models incorporating mHealth with in-person health care could improve the overall effectiveness and sustainability of CVD prevention and management [29].

Challenges and barriers to the implementation of mHealth

This use of mobile and wearable health devices has great potential in the prevention of CVD. However, like any other emerging technology, the implementation of these systems is beset with challenges and barriers that must be thoroughly addressed to fully realize their clinical utility. Patient populations, particularly older patients, have voiced concerns that the lack of physical interactions with healthcare providers is a significant barrier to mHealth adoption [36]. An argument for face-to-face consultations cannot be overlooked, as they play a crucial role in developing doctor-patient relationships and the detection of clinical indicators relevant to disease progression and treatment efficacy. These signals may go unnoticed in the absence of direct, in-person assessments. Additionally, there have been questions raised over the accuracy of the data they collect. Furthermore, their use is currently limited by the need for patients to be engaged in data collection over a long duration in order to collect the most reliable data to improve patient outcomes [37]. Therefore, successful integration of mobile and wearable devices relies on the involvement of healthcare professionals in the design process to ensure that service delivery meets the needs of all stakeholders involved [38].

Healthcare providers have expressed growing concerns regarding the regulation and clinical efficacy of mHealth applications. While developers frequently report optimistic outcomes, the existing literature lacks robust, evidence-based research that validates their effectiveness in real-world clinical practice [39]. Likewise, there is a noticeable absence of a standardized regulatory framework that establishes guidelines and benchmarks for the governance and quality assurance of mobile and wearable health technologies [40]. This challenge is exacerbated by regional variations in standardized healthcare practices, which complicate the development of a cohesive and unified framework. Without these frameworks, incorporating these devices into standard health services is hypothetical and unreliable [25,41].

Pre-established healthcare systems pose considerable challenges to their implementation despite the benefits. Healthcare providers exhibit resistance to change due to worries about patient retention, insufficient training on the application of mobile and wearable devices, and uncertainties regarding medical responsibility [36]. Moreover, private healthcare systems are susceptible to the prioritization of financial factors over service quality, negatively impacting patient outcomes, as cost considerations often take precedence over optimal care, limiting their effective utilization [42]. However, hindrances also reside in a rudimentary regulatory foundation, unequal access to electronic devices, and disconnected healthcare frameworks. Despite the skilled public health workers and organized programs, the access and durability of mHealth results have also been constrained by infrastructural and systemic obstacles [41].

Although continuous monitoring grants patients constant access to their medical data, it poses risks of misinterpretation or generation of inaccurate health information, consequently leading to unnecessary anxiety, prompting excessive and potentially unwarranted doctor visits [32]. Currently, mHealth and wearable devices have limited interoperability with existing electronic health record systems [36]. Integrating unverified patient-generated data into these information systems risks compromising the reliability of clinical data, which includes unwarranted procedures, augmented accountability, prolonged medical attention, and diagnostic error [24,25,41,43], as this has been built upon extensively researched, evidence-based protocols designed to ensure diagnostic accuracy and standardized patient care.

Cost-effectiveness of mHealth in CVD prevention

Numerous studies have shown that mHealth interventions can be a cost-effective approach to managing CVD risks. A systematic review of decision-analytic models assessed the cost-effectiveness of digital health interventions (DHIs) in CVD management. This review emphasized that DHIs could result in substantial health benefits at a reasonable cost, especially when aimed at high-risk groups. Nonetheless, the success of these interventions depended on their design and implementation [44]. A systematic review of decision-analytic model-based cost-effectiveness analyses supports this finding, as all, including DHIs, were cost-effective in terms of CVD management [33].

In another evaluation, a systematic review of decision-analytic models was performed to assess the cost-effectiveness of DHIs for CVD management. The effectiveness of the described DHIs varies, and although some DHIs present reasonable cost-effectiveness ratios, results are inconsistent across interventions and populations targeted. Such variation underscores the importance of tailored economic evaluations when considering the deployment of DHIs [45].

Changes in healthcare consumption patterns have been linked to the use of mHealth applications for CVD self-management. Research suggests that users of such apps may have fewer clinical visits, as they can better follow their health status and intervene early if problems arise. This shift has the promise of not only enhancing patient engagement but also lowering overall healthcare expenditures. Furthermore, there is consensus on the need for more comprehensive economic analyses, despite individual studies demonstrating the potential cost savings of mHealth interventions. A comprehensive assessment of mHealth technologies’ utility in CVD prevention needs to incorporate quality-adjusted life years, lifetime cost savings, and broader economic effects. This approach ensures that mHealth investments are underpinned by measurable health impact and return on investment [45].

A study showed that digital therapeutics for home-based cardiac rehabilitation are an exceedingly cost-effective rehabilitation strategy in comparison to conventional home-based cardiac rehabilitation [46,47]. As mHealth applications are increasingly integrated into healthcare delivery systems in various socio-economic and cultural settings around the world, there is a growing pool of economic evidence to support their use. A study suggests that, in individuals with heart failure who were unable to attend ambulatory rehab, telehealth-based therapy was not only viable but also possibly budget-friendly. Also, enhanced compliance due to adaptable timing, reduced load on hospitals, and a decrease in travel costs aided in a more streamlined distribution of medical care supplies without undermining patient results [47].

Long-term sustainability and scalability

Long-term sustainability and scalability of mHealth interventions depend on multiple factors, like technological practicality, user engagement, economic viability, and ease of integration into healthcare systems. The first factor for sustainability and scalability is financial and economic viability; hence, mHealth must display long-term financial benefits when compared to traditional CVD prevention methods (for instance, reduced hospitalizations). Additionally, they require sustainable funding sources, like public health initiatives, insurance reimbursements, government policy schemes, as well as private-sector investments [48]. Technology should be reasonably priced, which increases ease of scalability and decreases maintenance expenses. Furthermore, accessibility increases by ensuring that internet access and affordable smartphones in low-resource regions are imperative. Leveraging SMS-based interventions in limited internet regions increases scalability [6].

The second factor regarding the sustainability and scalability of mHealth is technological considerations. A key factor of interoperability is whether these mHealth apps can smoothly incorporate electronic medical records (EMRs) and mobile devices (e.g., smartwatches) [23]. The mHealth applications also require constant updates and improvements to keep up with security threats, software updates, and meeting the requirements of updating clinical guidelines. Lastly, mHealth applications must comply with the standards of the Health Insurance Portability and Accountability Act (HIPAA), the Food and Drug Administration (FDA), and other regulatory organizations [49].

The third factor concerning sustainability and scalability is user engagement and behavior change. This is judged based on how flexible the mHealth interventions are, to become personalized to individual risk profiles and medical backgrounds. Moreover, mHealth interventions' sustainability will be judged based on their gamification and incentives [17]. For example, interventions providing reminders or behavioral nudges to increase long-term user engagement. Also, health literacy is key; for instance, integrating simplified interfaces and multilingual options to improve user-friendliness throughout diverse populations [23].

**Figure 3:**
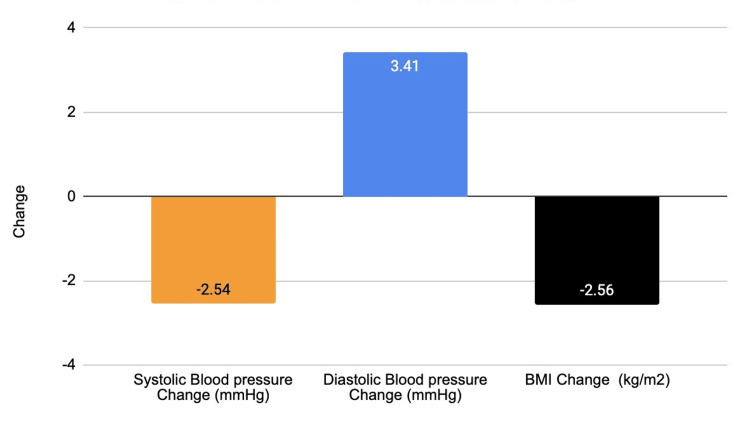
Effect of intervention on health metrics This image denotes the changes in blood pressure (mmHg) and body mass index (BMI) (kg/m²) with mobile health (mHealth) intervention. Image credit: Owais M. Ahmad

A great example of the sustainability of mHealth intervention was a study conducted by Grupo de Investigación en Salud Móvil en América Latina (GISMAL) involving 212 individuals. The outcomes established in the study were systolic and diastolic blood pressure levels, hypertension incidence, and body mass index (BMI) changes. While the intervention group did not display changes in systolic (-2.54 mmHg, 95% CI: -8.23 to 3.15) and diastolic (3.41 mmHg, 95% CI: -0.75 to 7.57) pressure compared to the control group, BMI changes showed significant reductions in BMI (-2.56 kg/m², 95% CI: -4.46 to -0.66) [50]. Additionally, individuals who received more than 50% of motivation during the past year demonstrated benefits that resulted in large reductions of BMI and body weight [51]. Figure 3 highlights the effect of intervention on health metrics.

Ethical and legal considerations in mHealth for CVD prevention

The integration of mHealth technologies into CVD prevention has the potential to revolutionize healthcare delivery in the 21st century; it can offer unprecedented opportunities for preventative, patient-centric care, as well as empower patient autonomy in the management of their health condition(s) [52,53]. However, the rapid adoption of these technologies has raised serious ethical and legal considerations that must be addressed to ensure their equitable and responsible use in the healthcare sector.

A major concern with mHealth applications is the fact that these applications collect sensitive personal information. This information must be protected against data breaches and unauthorized access and distribution [54]. To ensure confidentiality and trust, regulations such as the General Data Protection Regulation (GDPR) in Europe, HIPAA in the United States, as well as FDA digital health program regulations and policies, must be adhered to [55].

The mHealth user (patient) must be thoroughly informed regarding what data is being collected and stored, how it will be used and distributed, including the potential risks associated with using such a healthcare application. Patients should provide consent freely, in a language they understand. The term "patient activation" refers to an individual's knowledge, skill, motivation, and confidence in managing their health and healthcare [54]. Patients should be sufficiently "activated" such that the usage and implementation of the mHealth application foster an environment of trust and shared decision-making between the user and healthcare practitioner (HCP) [54].

Obvious ethical considerations arise when one considers socioeconomic barriers, geographic location (e.g., low- and middle-income countries), and digital literacy affecting the ability of users to fully and effectively utilize mHealth services [56,57]. It is essential to address disparities in access to such technology [35,58].

It is paramount that the health information provided by these applications is evidence-based, accurate, and reliable [59]. There are concerns regarding the reliability of the machine learning models that are used and the genuine risk of biased data affecting outcomes, which can lead to problematic issues in the decision-making process [54,58,60]. The FDA, in initiatives such as the Digital Health Program, issues directives that mHealth applications must undergo continuous validation and meet the appropriate regulatory, legal, and medical approval before allowing mainstream design, implementation, and adoption by users and HCPs [2].

A lack of standardization amongst different mHealth applications can lead to challenges in how data can be effectively shared across healthcare systems [61]. A major concern with deep learning (used in the development of mHealth applications) is its "black-box" nature, which makes it difficult for developers and HCPs to understand how exactly it operates and generates its output [3]. At present, there is no "one size-fits-all" deep learning model that is used in the architecture of different mHealth applications [62]. This raises issues regarding the continuity of care and interoperability between different healthcare systems [59].

Ideally, mHealth applications should be developed in collaboration with end-users (patients) [2,44,55]. When mHealth applications are prescribed by HCPs, patients may be more motivated to utilize them, versus when they simply download an application on their own, essentially enhancing patient engagement and adherence. In addition, this collaboration can potentially hold developers and healthcare providers accountable for the effectiveness and safety of mHealth interventions.

The ethical and legal considerations surrounding mHealth are multifaceted and require a collaborative approach amongst all stakeholders [57]. By prioritizing and addressing the above-mentioned points, stakeholders can harness the transformative potential of mHealth as a valuable digital tool in CVD prevention, whilst safeguarding patient rights and embracing the digital health revolution.

Future directions and innovations

A key area of innovation in the field of mHealth is AI. AI is most prominent, which is a handy tool for analyzing real-time data from EMR, wearables, and lifestyle inputs to produce a dynamic CVD risk evaluation and management plan. AI can also assist with its features, such as chatbots and virtual assistants, which can generate a custom lifestyle recommendation based on a patient’s risk factors and health background [4]. Additionally, AI, associated with ECG analysis (e.g., Apple Watch or KardiaMobile), is a great tool for the detection of Afib and arrhythmia before symptoms develop [63,64].

**Figure 4:**
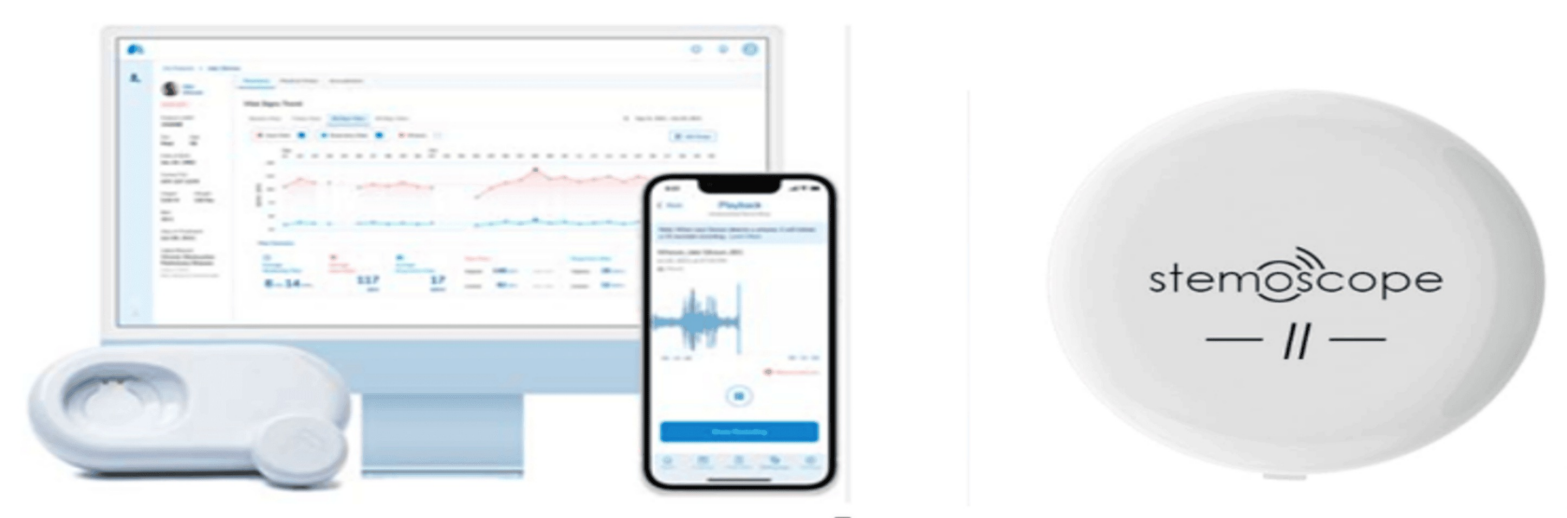
Image of wearable electronic stethoscope called Stemoscope II (left to right) These wearable devices need a display, such as a smartphone or laptop, which will allow effective communication with the patient. Image credit: This figure has been adapted from an open-access article [1], distributed under the terms of the Creative Commons Attribution-NonCommercial-NoDerivatives 4.0 License (CC BY-NC-ND 4.0) (https://creativecommons.org/licenses/by-nc-nd/4.0/).

A fascinating advancement in mHealth has been wearable stethoscopes, which are promising to revolutionize cardiac care. Wearable stethoscopes encourage remote patient care, decrease hospital visits, and supplement early intervention. However, a few drawbacks exist, like the accuracy of the wearable stethoscope, which has data security concerns and requires constant, stable connectivity for functioning [1]. Digital biomarkers are an imperative marker and key area of development for the future. These are involved in the continuous monitoring of heart rates, blood pressure, and sleep quality. Another key marker is non-invasive glucose, which can be quite beneficial for diabetic patients. Advancements are being carried out on smart clothing and biosensors, which are ECG-integrated shirts that can provide continuous cardiovascular monitoring [18]. Figure 4 shows the devices that allow effective communication with patients. 

Another area of development is remote cardiac rehabilitation. mHealth applications are now advancing by generating customized rehabilitation exercises and diet plans. Furthermore, telehealth enables engagement with specialists and medical follow-ups [18]. Voice assistants are another field of development in mHealth. For instance, Alexa, Google Assistant, and Siri are now offering heart health reminders, which are integrated with their respective medical applications. mHealth technologies are advancing, and their role in CVD prevention is evolving. Future developments will be focused on AI, user-friendliness, and enhanced integration into healthcare systems [17]. Table 2 outlines the technologies and their role in CVD prevention.

**Table 2:** Overview of impact of wearable devices on CVD prevention Table credit: This table was created by Owais M. Ahmad on Microsoft Word using the information from the reference cited [1]. AI, artificial intelligence; ECG, electrocardiogram; SVA, supraventricular arrhythmias; Afib, atrial fibrillation; CVD, cardiovascular disease

Future Innovative Technologies	Impact on CVD Prevention
AI	Smartwatches associated with AI have great features of ECG monitoring and analysis; hence, they allow for earlier detection of SVAs and ventricular arrhythmias. These are great tools to detect arrhythmias prior to the symptom onset.
Wearable Stethoscope	These devices allow for remote and continuous monitoring by providing real-time audio analysis of heart sounds. Moreover, this allows for early detection of abnormalities and provides time to plan for interventions of diseases like Afib, valvular heart disease or heart failure. Additionally, this provides patients with patient empowerment, as they are more informed about their cardiovascular health condition.
Smartwatches	Smartwatches are also a great tool to continuously monitor heart rate, blood pressure, and some devices also help with the measurement of blood oxygen. In addition, they are a great tool for physical activity tracking, for example, calories burned, steps taken, and exercise duration. Furthermore, a great feature of smartwatches is sleep quality monitoring. These watches help to monitor sleep duration and provide sleep scores, which help patients better monitor their sleep health.
Voice Assistants	Voice assistants remind patients when to take medications and monitor their blood pressure. They are great tools to track the amount of physical activity, meals consumed, and water intake. These tools help to empower patients to avoid back lifestyle choices and stick to a healthy diet and lifestyle to better help in CVD prevention.

Limitations

In this review, we have identified numerous limitations that should be addressed before establishing a conclusion. Firstly, a systematic review methodology was not utilized; hence, there is a risk of leaving out articles that contain relevant information. Unlike systematic reviews, narrative reviews do not adhere to a strict methodology for article selection and data evaluation. Therefore, this may lead to selection bias, as only particular studies are selected based on the authors' decision.

Secondly, there is a lack of quantitative analysis; therefore, this limits the ability to formulate conclusions about the effectiveness of mHealth and wearables in CVD prevention. The presence of quantitative data would have enabled the extrapolation of data and the establishment of a more objective conclusion regarding CVD prevention using mHealth. Another limitation is the heterogeneity of the study designs used in this narrative review. Each study on mHealth and wearable devices differs in methodology, target population, intervention types, and outcomes measured. Thus, this variability in study design makes it difficult to generate an evidence-based conclusion.

It is significant to note that epidemiological factors could limit the ability to generalize findings. Furthermore, individuals from less developed countries (LDCs) would have different cardiovascular risk factors from individuals in more developed countries (MDCs). Also, individuals from LDCs would have less access to mHealth, in comparison to individuals in MDCs; hence, CVD prevention outcomes would differ across different populations.

Another bias that should be addressed is publication bias. With relevance to our review, studies showing positive CVD prevention results from the usage of mHealth devices are more likely to be analyzed. Hence, this causes an overjudgment of benefits and underrepresentation of the challenges associated with the role of mHealth in the prevention of CVD. A narrative review can become outdated due to rapid advancements in mHealth and wearable technology. Thus, it is imperative to account for updates in the evolving technology industry. 

To improve the limitations mentioned above, investigations using standardized mHealth devices, corresponding to standardized CVD prevention outcomes, would be very beneficial in establishing more founded conclusions. 

Finally, future research should target standardized methodologies and incorporate larger, ethnically diverse populations to improve the reliability and authenticity of clinical conclusions. 

## Conclusions

A key advancement in modern healthcare is the evolving field of mHealth and wearable devices in CVD prevention. These technologies include smartwatches, patches, wearable stethoscopes, and biosensors, which offer continuous monitoring of key cardiovascular metrics like blood pressure, ECG, and heart rate, hence enabling early diagnosis and intervention. For example, the RITMIA app-based mHealth has been proven superior to classic 12-lead ECG in diagnosing Afib. In addition, mHealth is seen to aid in improving weight control and adherence to medications, although it shows no significant difference in SBP readings compared to controls. However, a major benefit of wearables is their integration with traditional healthcare, allowing data to be shared between patients and healthcare providers.

Moreover, achieving sustainable funding sources, such as for public health initiatives, and a user-friendly interface are central to long-term sustainability and scalability. Presently, mHealth trends incorporate AI and telemedicine for more fine-tuning of treatment personalization. Yet, despite these benefits, elements of legal and ethical issues, such as data privacy and compliance with regulatory authorities, present barriers to its widespread use. Moreover, the implementation of mHealth technologies among diverse global populations yields variable effectiveness, depending on the level of digital literacy and disparities in access to technology in resource-constrained countries. Most specifically, the health engagement considered in mHealth and wearables provides an enhanced device to not only maximize early detection but also to involve patients in their own care in a cost-effective manner, directed towards prevention. However, overcoming ethical, regulatory, and accessibility challenges will be important for broad implementation and sustained success. Further studies must focus on optimizing these technologies to enhance their incorporation into conventional healthcare systems and stimulate equitable accessibility among all different populations.
